# A cross-sectional study investigating malaria prevalence and associated predictors of infection among migrants to a newly established gold mining settlement in the Gambella Region of Ethiopia

**DOI:** 10.1186/s12936-024-05117-4

**Published:** 2024-09-30

**Authors:** Natasha Glendening, Werissaw Haileselassie, Ming-Chieh Lee, Behailu Taye, Yonas Alemu, Ayele Belachew, Wakgari Deressa, Guiyun Yan, Daniel M. Parker

**Affiliations:** 1 Joe C. Wen School of Population and Public Health, College of Health Sciences, University of California at Irvine, Irvine, CA 92697 USA; 2 School of Public Health, Addis Ababa University, Addis Ababa, Ethiopia; 3 Department of Biology, College of Natural and Computational Sciences, Mettu University, Mettu, Ethiopia; 4 Aklilu Lemma Institute of Pathobiology, Addis Ababa University, Addis Ababa, Ethiopia; 5 Department of Microbiology, Immunology and Parasitology, College of Health Sciences, Addis Ababa University, Addis Ababa, Ethiopia

**Keywords:** Gold mining, Ethiopia, Migration, Extractive settlements, Malaria

## Abstract

**Background:**

Malaria is a major disease burden in Ethiopia. Migration can influence malaria transmission dynamics, with individuals relocating from malaria-free highland regions to malarious lowlands potentially facing elevated risks of contracting malaria. Migrants may find it difficult to protect themselves against malaria and have limited access to diagnosis or treatment. Settlers in gold mining sites are one type of migrant and are often neglected in malaria research yet may have particularly high malaria risk. This study was a malaria prevalence survey among settlers in a new gold mining settlement in the highly malarious Gambella Region, Ethiopia.

**Methods:**

n = 590 people were surveyed for demographic information and their knowledge and practices of malaria. Participants were tested for malaria using rapid diagnostic tests and microscopy. Using logistic regressions, the influence of demographic characteristics on malaria infections and bed net access were analysed. A sub-sample of participants was interviewed to comprehend settlement living conditions and healthcare accessibility.

**Results:**

The overall prevalence of *Plasmodium falciparum* was 37.5% (CI 32.4–42.3%). Young children were most likely to have malaria, with individuals aged 15–24 having 67% lower odds (aOR: 0.33; CI 0.13–0.86) of infection compared to those aged 1–4 years old. Meanwhile, those age 25-plus had 75% decreased odds of malaria infection (aOR 0.25; CI 0.10–0.65). Individuals with bed nets had ~ 50% decreased odds of testing positive for falciparum malaria than those reporting having no bed net (aOR: 0.47; CI 0.22–0.97). Individuals who relocated from low elevation with high malaria test positivity rate areas were more prone to testing positive for malaria, as were those residing in densely populated households with multiple malaria cases. Conversely, individuals from higher elevations with low malaria test positivity rates, and those living in households with 5–10 occupants and < 2 malaria infections, were more likely to possess bed nets.

**Conclusions:**

This gold mining settlement provides an example of an oft-neglected atypical community where malaria is a significant, but under-addressed, health problem. Within this community, future interventions focused on distributing bed nets, particularly to larger households and those with children, have great potential to alleviate the malaria burden. Efforts should also be made to provide affordable, and accessible, early diagnosis and treatment.

**Supplementary Information:**

The online version contains supplementary material available at 10.1186/s12936-024-05117-4.

## Background

Malaria continues to be a major burden of disease in Ethiopia. Most cases are caused by infection with *Plasmodium falciparum* parasites, but *Plasmodium vivax*, *Plasmodium ovale*, and *Plasmodium malariae* are also present. In 2022, there were an estimated ~ 5.1 million malaria cases in Ethiopia and approximately 10,570 deaths [1]. Despite government commitment to malaria elimination and much progress to reduce malaria incidence and deaths from malaria year on year, Ethiopia remains among the top 20 countries in the world for malaria cases and deaths from malaria [1].

One factor affecting malaria dynamics in Ethiopia, and one that will need addressing in order to fulfill the Ethiopian government’s malaria elimination goals, concerns internal migration. Migration from highland areas to lowland areas has been highlighted as a potential public health problem in Ethiopia [2, 3]. Typically, in Ethiopia, malaria incidence has a heterogenous distribution along elevation lines, with lower incidence at higher elevations and higher incidence at lower elevations (see Supplemental Fig. 1) Although, it should be noted that elevation is not the only factor affecting malaria incidence. However, numerous factors have increased migration from relatively low-incidence and high population density highland areas of Ethiopia, to the more malarious lower elevation areas, partially fueled by economic opportunities in lowland areas [4–7].

Several studies have shown that migrants from low or no incidence highland areas of the country traveling for seasonal work in high incidence lowland areas have increased risk for malaria infection compared to non-migrants [8–14]. This has the potential to increase malaria incidence in lowland areas, as well as to spark disease outbreaks in migrants’ low incidence origin communities from importation of malaria cases among these communities [3].

One reason for the apparent association between migration and malaria incidence may be due to increased exposure to malaria mosquito vectors in migrants’ work environments which may also increase their malaria risk compared to non-migrants [8, 13]. Another explanation may be immunological naivety for malaria among those originally from low incidence areas in the highlands of Ethiopia [4, 12, 13, 15, 16]. Additionally, migrants are often of a low socioeconomic status and may live in poor housing units that can exacerbate exposure to malaria vectors and increase risk of malaria infection [8, 11, 13]. Studies have also found that internal migrants in Ethiopia often have more limited options for malaria prevention and treatment in their new communities [8–10, 12–14].

One type of migrant that is common in Ethiopia, but often neglected in public health research and control initiatives, are settlers—those that have migrated from other communities to form a new settlement that was not previously established. At the start of a new settlement, everybody in the population there is a migrant that has moved, even if it was from nearby areas. This type of migrant is the focus of this study. Settlers often share similar experiences and risk factors to other migrants, such as having low socioeconomic status and poor housing conditions, but these issues can be exacerbated due to the lack of established community resources [17–20]. For example, settlers that have moved to newly emerging settlements will not have access to pre-arranged sleeping quarters or malaria prevention and treatment options that companies occasionally provide, in the same way that government and company sponsored migrants might do. Settlers living in these emerging settlements, especially those that are established around extractive industries, can be simultaneously more vulnerable to many health issues [21–30] including malaria [18–20], whilst also experiencing poorer access to healthcare resources, even in comparison to other migrants [20].

## Objectives

This research investigates the burden of malaria and correlates of malaria infection in Lunga, a newly formed gold mining settlement, located in Gambella Region—one of the most malarious regions of Ethiopia [31]. The study aims to go beyond simply investigating the prevalence of malaria infections; it seeks to understand the factors that are associated with malaria infection within this unique population of settlers. Given the known association between migration from low-incidence highland areas and increased malaria risk, the potential risk factors were explored, including bed net access and demographic characteristics, that may influence malaria infections in this newly established settlement. The primary outcomes in the analysis are (1) *P. falciparum* malaria infections and (2) bed net access, with a focus on examining potential differences between demographic groups and understanding the underlying factors contributing to these outcomes.

## Methods

This study was a cross-sectional survey with both quantitative and qualitative components. Data were collected over a 2-week period in September 2022 by a trained research team.

### Study site

**Fig. 1:**
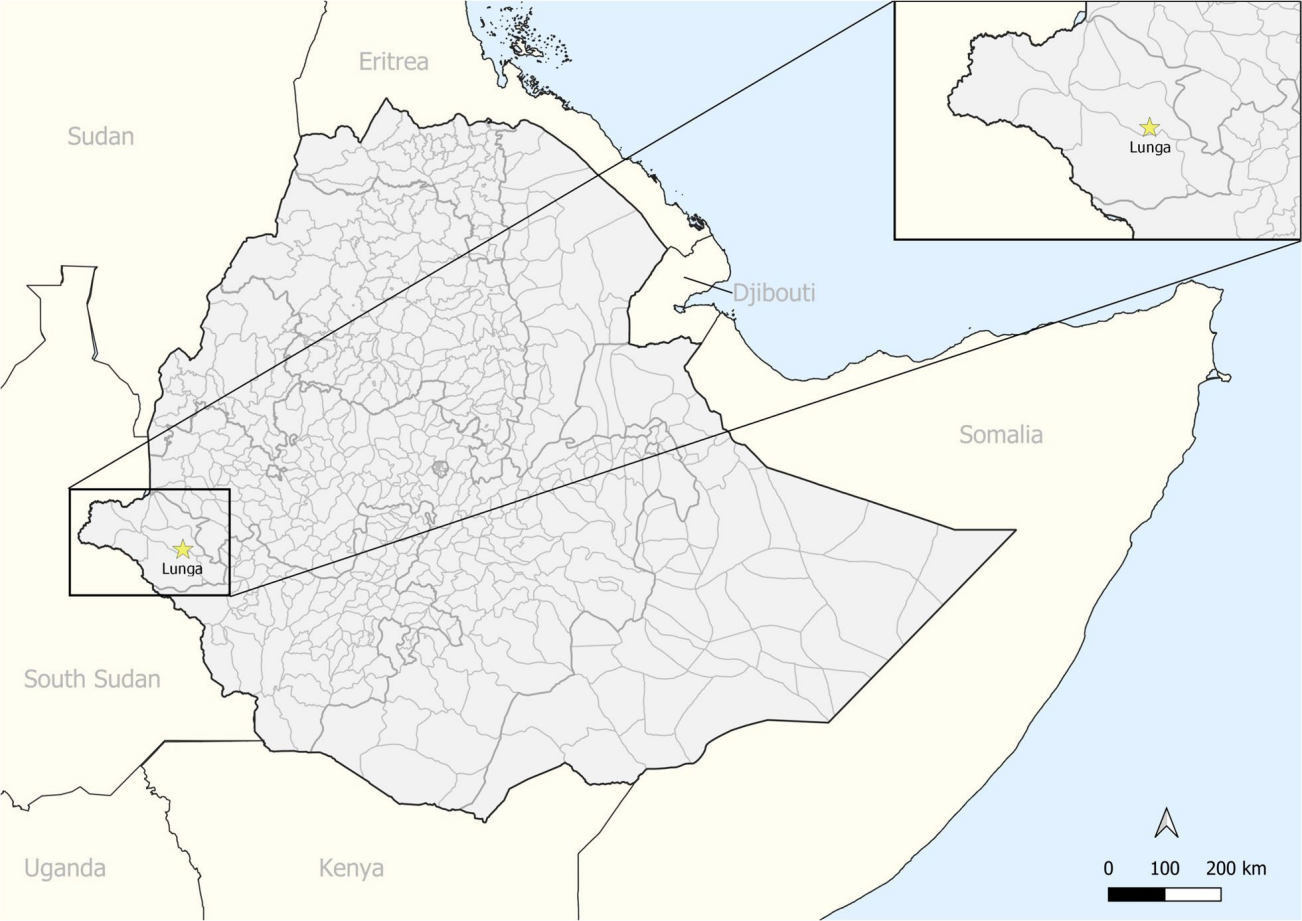
Location of study site in Ethiopia

Lunga is a newly emerging settlement formed around artisanal and small-scale mining (ASM) in the highly malarious Gambella region of Ethiopia (see Fig. 1 for location). Since around ~ 2016, migrants have started moving to Lunga from across Ethiopia to take advantage of the economic potential from gold mining there [32]. This has led to a rapid expansion of the population, with an estimated ~ 12,000 people in 2021, in what previously was a mostly forested area with limited accessibility [32]. Until recently (~ 2016), Lunga did not exist; thus, every individual in the study population is, by definition, a migrant or settler who relocated to this area to establish and expand this new settlement, even if they have moved from within the same region of Ethiopia.

### Data collection

#### Detection of malaria parasite infections

All participants were tested for malaria using SD Bioline Malaria Ag P.f/Pan rapid diagnostic tests (RDTs). With this RDT the antigen tested for *P. falciparum* is histidine-rich protein 2 HRP2 antigen and *Plasmodium* lactate dehydrogenase (pLDH) for other species. Blood samples (~ 300 μL) were also taken by finger prick, from consenting individuals for microscopy analysis to categorize infections, including by species. A certified expert microscopist from Addis Ababa University analysed the blood samples using WHO Giemsa staining protocols. Tentative results were then read by a 2nd microscopist and if there was a discrepancy between the two microscopists’ results, then a third microscopists reviewed the slide and their interpretation of the slide was accepted as the slide result. Individuals were then classified as positive for a particular species (*P. falciparum* or *P. vivax)* if they tested positive for that species through RDT and or microscopy testing.

#### Knowledge attitudes and practices survey

A structured Knowledge, Attitudes and Practices (KAP) of malaria survey was administered to consenting participants in each selected household to collect information on participant age, gender ethnicity, education level, their knowledge and practices with regard to malaria, and perceived health care availability in the local area. The KAP survey was informed and developed based on a pilot study in the same community in 2020 [32]. The questionnaire also included questions about the place of origin for settlers in the community and how long they had lived in the community (see Supplemental Fig. 2). The questionnaire was developed in English and delivered verbally by field workers to participants in a mutual language and answers were translated back into English before being written up using electronic tablets. The KAP survey was delivered to all participants, but parents answered on behalf of their children and so the analysis of the KAP questions were restricted to adults only.

#### Qualitative interviews

The main aims of the qualitative component were to understand the processes through which community members learned about Lunga, to develop a better understanding of the living conditions in Lunga, and to better understand the public health and healthcare situation in the community. Individual interviews were conducted face-to-face with a purposeful sub-sample of the study population in a local language and then translated into English. The sub-sample was selected using maximum variation purposive sampling, based on demographic characteristics, to be representative of the ethnicity, age, and gender makeup of the community. To help guide the interview, a semi-structured questionnaire was used for the interviews that focused on reasons for moving to Lunga; conditions of the community; access to health care, and personal experiences with malaria infections (see Supplemental Fig. 3).

### Sample size and sampling procedure

A single-stage random cluster sampling strategy was used, whereby each cluster was a household in Lunga. Households were randomly selected from a household registry of those living in Lunga. The research team went to each selected household and invited them to participate in the study. Once households were selected, every household member over 12 months of age was invited to participate in the quantitative portion of the study by the research team. A household could participate if at least one person was eligible for the study and consented to participate. The qualitative interviews were restricted to only those 18 years or older. For both the quantitative and qualitative portions, only those that considered Lunga to be their primary residence were eligible to participate. These criteria allowed for a focus on settler migrants rather than temporary and transient workers, as few research studies have specifically focused on settler communities in this area of Ethiopia before. However, the criteria may have excluded individuals who were in the settlement but did not claim to live there, from the analysis.

The sample size for the quantitative portion was determined with consideration for the primary outcome of the analysis, which was estimated P. *falciparum* prevalence. Given the clustered nature of data collection (with households as clusters) sample size calculations were adjusted in light of this, following Thompson’s approach [33]. Preliminary work in the setting led to an estimated mean household size of 5 people. Assuming that 50% of the population may have malaria infections, and assuming an intra-cluster correlation coefficient (ICC) of 0.03, this produced an estimated need for at least 84 households (clusters) for a total of 418 participants.

Qualitative work does not rely on necessary sample size calculations in the same manner as quantitative research [34]. As such, for the qualitative component, this study aimed for at least 30 community members to be recruited from the sample established in the quantitative portion and agreed to continue until saturation in the content of interviews was reached. A sample size of 30 individuals is on the high end of sample sizes for interviews in qualitative research [35]. In total, 31 community members were interviewed.

### Data analysis

#### Quantitative analysis

**Fig. 2:**
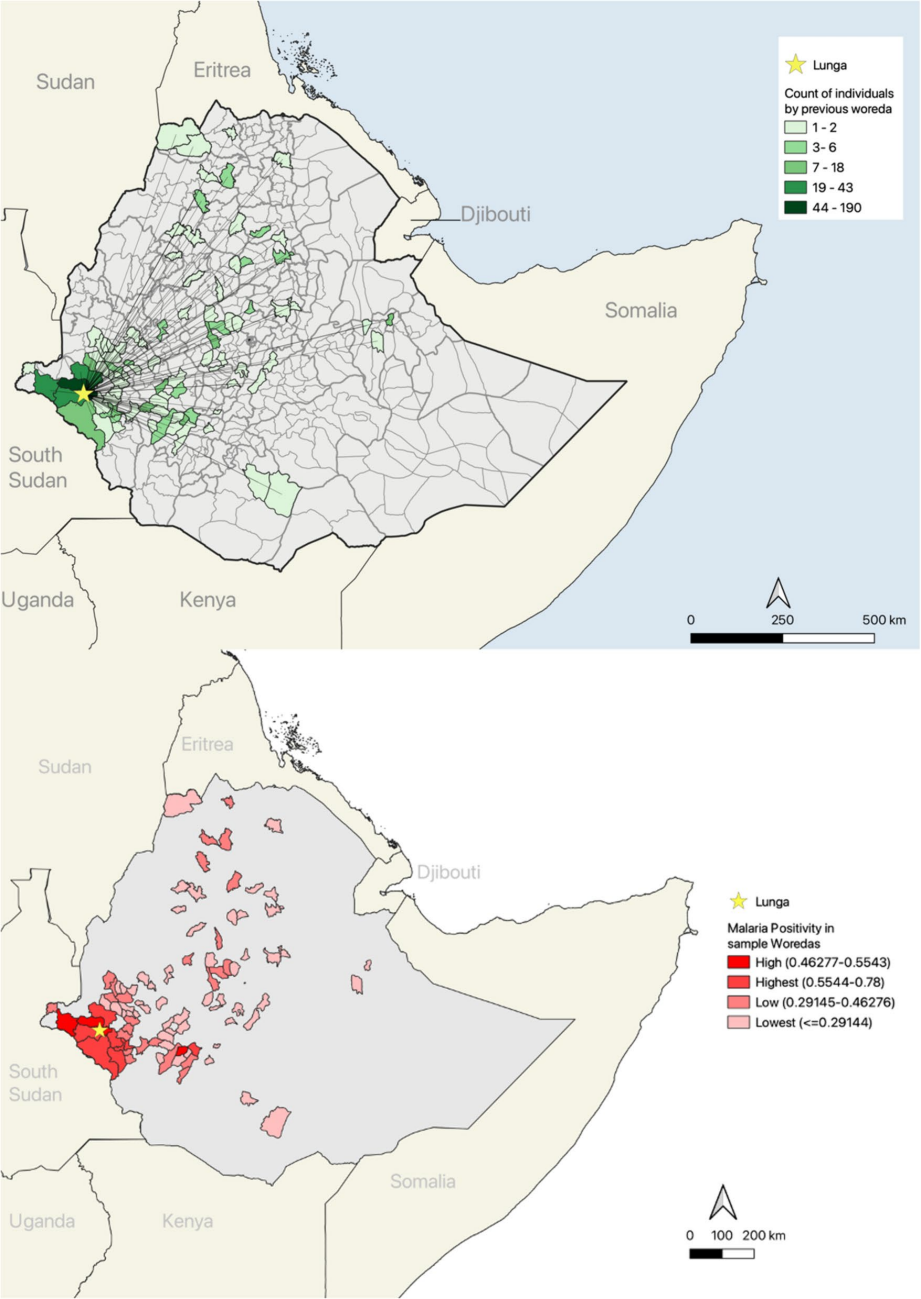
**A** Map of places of origin (woredas) from which Lunga settlers moved. **B** Malaria test positivity in woredas from which Lunga settlers moved

The *woredas* (i.e., districts) from which a person lived immediately prior to moving to Lunga were identified from the data collected in the survey which asked participants to self-report the village, woreda and region names of their prior location. These woredas, along with the GPS coordinates for the study site (Lunga), were then identified on a map of Ethiopia using QGIS 3.22.1 software. The geographic centroids (central location) of each woreda were calculated using the centroids function in QGIS. Next, a distance matrix was utilized to generate the straight-line distance from each of the centroids to the study site. This allowed us to produce a map (Fig. 2A) indicating the number of people that had lived in each woreda prior to moving to Lunga, and an estimate for how far these locations were from Lunga.

Using QGIS (3.22.1) an estimate for the average elevation of each woreda a person had lived in immediately prior to moving to Lunga was produced, which was a variable in the models. The mean elevation of the woredas a person had lived in was calculated by applying the zonal statistics function in QGIS to a raster digital elevation model (DEM) file of elevation for Ethiopia.

The malaria test positivity for each woreda a person had lived in immediately prior to moving to Lunga was calculated, which is another variable in the models. Test positivity was calculated as the number of positive malaria tests divided by the total number of malaria tests in each woreda. The data were acquired from the Ministry of Health and were aggregated from malaria incidence reports from local public health agencies, with the original data collected from each woreda’s health facilities using passive case detection. These data were from 2021 and 2022 and was calculated as a mean across 2-years (2021 and 2022) if both years were available, and otherwise used the single year of data. A map in QGIS was produced to graphically display the malaria test positivity for each woreda a person in the sample had moved from (Fig. 2B).

Generalized additive models (GAMs) with binary distributions and a random intercept for household were used to test for the odds of testing positive for *P. falciparum* as well as for the odds of owning a bed net. An individual was coded as owning a bed net if they reported having a bed net for personal use. As such, a person who lives in a household with someone else that has a bed net might not be categorized as having a bed net if they did not report having one for personal use. A random intercept for households in the GAMs were used, with multiple individuals being attributed to a single house, in order to adjust for the clustered nature of the data (i.e. the survey sampled households rather than individuals at random). The use of GAMs also allowed for the incorporation of smoothed spline functions for continuous variables and interactions in the regressions.

The regressions included both individual and household-level variables (Table 1). Variables included age, gender, self-reported bed net ownership, the elevation of the location from which an individual had migrated, the test positivity of the area from which an individual had migrated, the duration of time the person had lived in Lunga, household size, and number of other household members testing positive for malaria (Supplemental Table 1). In order to test for potential effect modification of household level variables on individual factors, the models were first run with only individual-level variables, and then subsequently with both individual-level and household-level variables. Changes to the coefficients for individual level variables after the inclusion of household level variables could indicate effect modification.

**Table 1:** Model results from generalized additive logistic regression mixed models

Variable	n	n RDT +	n Microscopy +	N PF + (%)	N (Bed net) (%)	Model 1A (Odds of testing positive PF—individual covariates)	Model 1B (Odds of testing positive PF—household covariates)	Model 2A (Odds of having access to a bed net—individual covariates)	Model 2B (Odds of having access to a bed net—household covariates)
*Age group*									
1–4 (reference)	48	22	8	22 (46)	7 (22)	–	–	–	–
5–14	43	19	10	23 (55)	3 (9)	0.74 (0.25–2.15)	0.95 (0.29–3.12)	0.24 (0.04–1.30)	0.32 (0.05–1.85)
15–24	248	92	47	100 (41)	28 (13)	0.50 (0.21–1.16)	0.33 (0.13–0.86)	0.41 (0.15–1.17)	0.53 (0.18–1.59)
25 +	250	80	32	83 (34)	21 (10)	0.40 (0.17–0.93)	0.25 (0.10–0.65)	0.35 (0.12–0.99)	0.43 (0.14–1.29)
*Gender*									
Female (reference)	255	93	37	99 (39)	34 (15)	–	–	–	–
Male	335	120	60	129 (40)	25 (9)	0.89 (0.59–1.35)	0.68 (0.42–1.1)	0.60 (0.32–1.10)	0.75 (0.40–1.43)
*Bed net*									
No (reference)	449	173	77	187 (42)	–	–	–	–	–
Yes	59	15	9	14 (24)	–	0.47 (0.24–0.91)	0.47 (0.22–0.97)	–	–
AIC (Akaike Information Criterion)						587.0449	538.9278	329.5844	307.1608
n				228		448	448	450	450

Table Information: Model 1: Logistic regression for odds of testing positive for falciparum malaria; Model 2: Logistic regression for odds of having a bed net. The models included spline functions for time since moving to Lunga (Supplemental Figs. 4b, 5c, 6b, 7c), an interaction between the elevation of previous residence and malaria test positivity in that previous residence (Supplemental Figs. 4a, 5b, 6a, 7b) and an interaction between the number of people living in a household and the number of people with malaria infections in that household (Supplemental Figs. 5a, 7a). Model A for both models included only individual-level covariates while Model B also included the household level interaction term (number of household members and number of household members with malaria infections)

The results from the GAMs are presented as model-adjusted odds ratios (aOR) and their confidence intervals for linear predictors and as plots of spline functions for the continuous variables that were modeled as splines. The model building procedure and model diagnostics suggested that household size and number of other household members with infections were best incorporated as an interaction term. Likewise, the study found that the variables for elevation of the place of origin and the test positivity of the place of origin should also be specified as an interaction term (see Supplemental Fig. 1—these two variables are inversely related to each other).

#### Qualitative analysis

The audio recordings of the interviews were transcribed and translated into English. Interview transcriptions were processed and handled by Atlas ti 7 version 7. 5.16 Software. The transcripts were then typed in Microsoft Word. Using grounded theory, a code book was created after an initial reading of the transcripts (based on key themes that emerge through the interviews). The transcripts were then coded and analysed using a thematic analysis approach. A sample of quotes were then chosen by the authors to best represent the themes that emerged through the analysis.

### Ethical approval

IRB approval was granted by the College of Health Sciences at Addis Ababa University. Verbal and written informed consent were required from individuals before participating in the study, participants could opt out of the blood sample collection and still participate in other elements (e.g. the KAP survey, rapid test, or qualitative interview). For children under 18 years of age, assent/consent for participation was granted by their parent or legal guardian. Individuals who tested positive during the survey were offered antimalarial treatment according to national treatment guidelines.

## Results

While most of the participants were from areas nearby Lunga (55%, n = 322, came from within Gambella Region), many came from relatively far away (farthest estimated distance = 850.73 km; Fig. 2A). Among participants, gold mining was the most commonly reported occupation, with 46.55% (n = 270) stating gold mining as their main occupation.

Out of a total of 590 people in the malaria survey, 34 people had no RDT results and 57 had no microscopy results. 213 were positive by RDT during the survey and another 26 were found positive by microscopy, leading to a total of 239 participants being positive for malaria infection (including both *P. falciparum* and *P. vivax* infections). Out of the 239 total malaria infections, 228 were positive for *P. falciparum* (including mixed infections), 70 were positive for *P. vivax* (including mixed infections), and 49 were positive for both *P. vivax* and *P. falciparum*. A further 11 people tested positive for malaria but for discordant species (*P. falciparum* and *P. vivax*) on RDT and microscopy testing. These 11 people were not classified as having co-infections for P. falciparum and P. vivax as there was no evidence of mixed infections within the same test (RDT or microscopy). They are however included in the *P. falciparum* and *P. vivax* figures. One individual tested positive for malaria but with no documentation regarding species, this individual was not included in the main analysis which was restricted to confirmed cases of *P. falciparum* only*.*

After accounting for the clustered design of the survey, an estimate of *P. falciparum* malaria prevalence (including those infections concurrent with *P. vivax*) of 37.5% (CIs 32.4–42.3%) was found. Since there were very few *P. vivax* infections in the data (8.8% of RDT samples), the analysis focused on *P. falciparum* malaria. Individuals with missing data were excluded from the analysis (n = 142 for Models 1A, 1B and n = 140 for Models 2A, 2B). However, as community members were generally enthusiastic about participating in the study due to free RDT testing and treatment, the sample size was actually much higher than necessary. As such, even after removing participants with partially missing data the final models still included more individuals than necessary. There was an association between age and odds of infection (see Table 1). In comparison to the youngest age group (1–4) those in the 15–24 age group had a 67% decrease in odds (aOR: 0.33; CI 0.13–0.86). Similarly, also in comparison to the youngest age group (1–4), those in the 25 + age group had 75% decrease in odds (aOR: 0.25; CI: 0.10–0.65) of testing positive for *P. falciparum* infections. There was no association between gender and odds of infection.

**Fig. 3:**
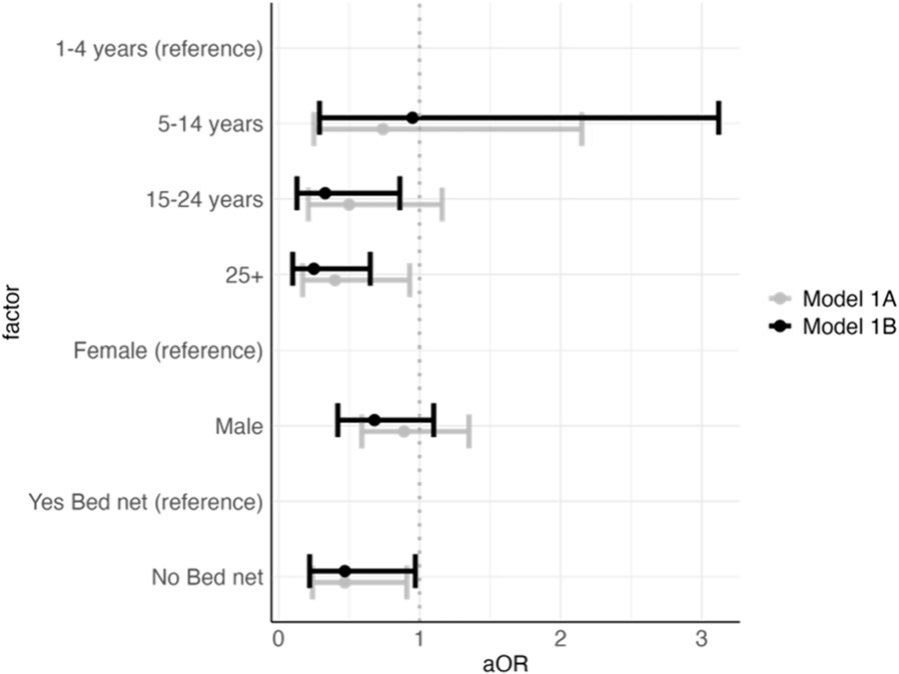
Model 1 Results: Odds of testing positive for falciparum malaria. **A** Model with only individual level predictors. **B** Model with household level predictors (household level variables were modeled as splines and are shown in Supplemental Fig. 5a–c)

Individuals who reported having a bed net also had decreased odds of testing positive for falciparum malaria (aOR: 0.47; CI 0.22–0.97). Figure 3 graphically displays the model results of the odds of a positive falciparum infection, with both individual and household level factors, as a forest plot. While many of the individuals who tested positive for malaria were from low elevation, high test positivity areas (many were from Gambella Region originally), the interaction term was not statistically significant (see Supplemental Table 3).

The interaction term for the number of people in a participants’ household and the number of people who tested positive in the household (excluding the person of reference) indicated that household crowding and living with others who had active infections was associated with increased odds of having an infection (see Supplemental Table 3). For example, odds of testing positive were high for individuals who shared a household with 10 or more other individuals and with 5 or more of those individuals having active infections (see Supplemental Fig. 5).

The study found no association between the duration of time a person had lived in Lunga and their odds of testing positive during the survey.

### Predictors of owning a bed net

**Fig. 4:**
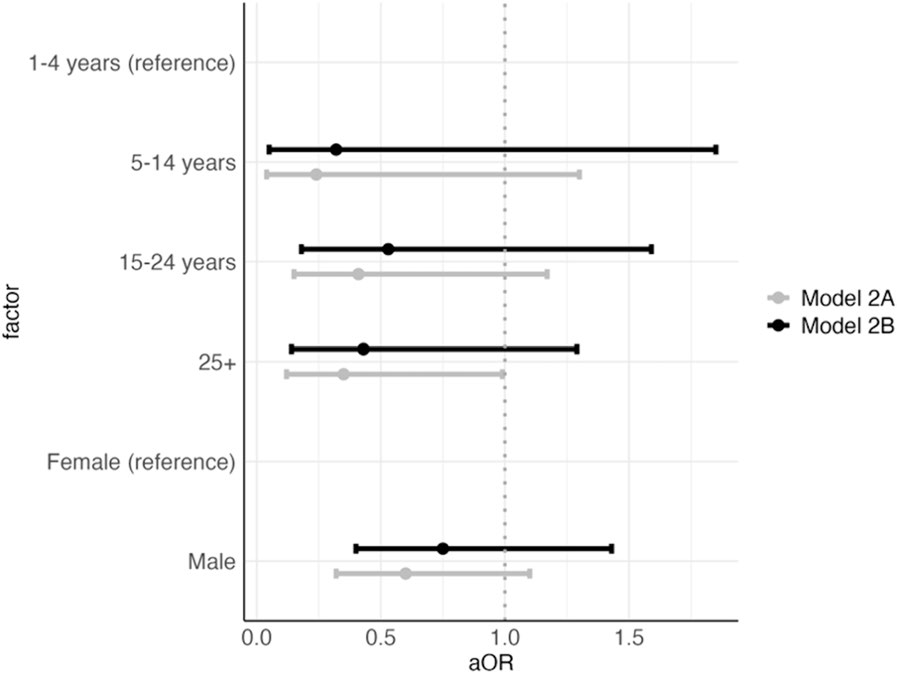
Model 2 Results: Odds of having access to a bed net. **A** Model with only individual level predictors. **B** Model with household level predictors (household level variables were modeled as splines and are shown in Supplemental Fig. 7a–c)

The model for odds of having a bed net with only individual level factors included suggested that the older age group (25+) had 65% lower odds of owning a bed net (aOR: 0.35; CI 0.12–0.99, see Table 1: Model 2A) Fig. 4 graphically displays the model results of the odds of owning a bed net, with both individual only level factors and household level factors, as a forest plot.

Most individuals with bed nets were coming from high elevation places that had low malaria test positivity, however the interaction term was not statistically significant in the model (Supplemental Table 4). Conversely, the interaction term for number of individuals in a house and the number of individuals in a house with a malaria infection, it indicated that individuals with households sizes of around 5—10 and with no or very few (< 2) other household members with a malaria infection were more likely to have bed nets (see Supplemental Fig. 7 and Supplemental Table 4). Individuals who lived in larger houses but with little-to-no malaria infections in the house were most likely to have a bed net.

### KAP results

The majority of people over the age of 18 knew of malaria (97.5%, see Table 2) and knew that malaria is caused by mosquito bites (89%). Age was negatively associated with knowledge that mosquito bites caused malaria (aOR = 0.95 CI 0.91–0.99, see Supplemental Table 5). Being a gold miner was also associated with 68% lower odds of knowing mosquito bites caused malaria (CI 0.12–0.85) compared with non-gold miners. Whereas being male was positively associated with knowledge that mosquito bites caused malaria (aOR 3.11 CI 1.23–7.86). Meanwhile, education was not significantly associated with knowledge of mosquito bites causing malaria.

**Table 2:** Knowledge of malaria among adults in the sample

Variable	Categories	Frequency (n)	Percentage (%)
Knowledge of malaria	Yes	429	97.5
	No	11	2.5
Knowledge of cause of malaria	Mosquito bites	425	89
Knowledge of who gets a serious case of malaria	Pregnant people	356	74.6
	Children under 5	160	33.5
Knowledge of sign/symptoms of malaria	Fever	409	85.7
	Feeling cold	283	58.8
	Headache	346	72.5
	Nausea and vomiting	163	34.2
	Dizziness	111	23.3
	Loss of appetite	56	11.7
	Body ache/joint pain	56	11.7
	Body weakness	80	16.8
Knowledge of malaria prevention methods	Bed net	409	85.7
	Mosquito repellent	23	4.8
	Spraying house with insecticide (IRS)	70	14.7

Nearly three quarters of adults in the sample also knew that pregnant people could develop a serious case of malaria, but only 1/3rd were aware that children under the age of five could also develop a serious case of malaria (see Table 2).

Most people could identify common malaria symptoms such as fever (85.7%), feeling cold (58.8%) and headaches (72.5%). Other symptoms such as dizziness (23.3%), loss of appetite (11.7%), body ache (11.7%) and body weakness (16.8%) were less commonly identified as malaria symptoms (Table 2).

In terms of knowledge of malaria prevention methods, 85.7% of people knew bed nets were effective to prevent malaria, but knowledge of mosquito repellent (4.8%) and IRS (14.7%) was much lower (Table 2). No variables were significantly associated with knowledge of bed nets as prevention methods against malaria (see Supplemental Table 5). Although, being male was negatively associated with knowledge of mosquito repellent as a prevention method against malaria (aOR: 0.26 CI 0.07–0.98, Supplemental Table 5).

Personal access to a bed net was low throughout the sample population (11.6%, Table 3). When asked why a bed net wasn’t used, 99% of people indicated this was due to not owning one. Among those that do have access to a bed net, 86.4% of people mentioned using it the night prior to the survey.

**Table 3:** Malaria prevention usage among all participants

Variable	Category	Frequency	Percentage (%)
Have personal access to a bed net	Yes	59	11.6
	No	449	88.4
Used a bed net last night (if own one)	Yes	51	86.4
	No	8	13.6
Reason for not using a bed net (if don’t own one)	Do not own one	95	99

#### Qualitative findings

Four main themes emerged from the interviews. The first theme had to do with why villagers had first moved to the location; another had to do with general living conditions in Lunga; another was more specifically focused on healthcare access and public health; and the last had to do with an apparent community support network.

#### Reasons for moving to Lunga

Most villagers interviewed explained that they had heard about the opportunities at Lunga through family, friends or acquaintances they met. Some had initially come to the Gambella Region or other mining opportunities and then moved to Lunga after hearing about the gold available.

*“I went to Abobo district for another work and I heard from people that there is gold in lunga. Then I decided to see this place.”*

*“I heard there was an opportunity for a gold business. I came and see it myself. I heard it from my friends.”*

“I heard there is a mining opportunity here in the media and from people at Gambella. I was one of the first people who come in here. It has have been 5 years.”

#### General living conditions in Lunga

Nearly all agreed that Lunga was an expensive place to live and that there were currently very little services available in the community including drinking water, schools and medical resources.

*“Everything is expensive in here. The food and all the basic needs are expensive. I think the gold mining exaggerated the living condition too. Its worse than the national condition, for example of you take drinking water, it’s not even purified water, they just collect from village and put it in a container then they sell by 10 or 15 birr [$0.18-0.26 USD]”.*

*“There are a large number of people in here but there is no medical care, water supply and schools are not sufficient when we compare with other towns”.*

#### Healthcare access and general public health

In terms of healthcare, most people mentioned that bed nets were difficult to find in the local area and required extensive travel to buy them in other big towns. Bed nets were also considered an unaffordable luxury for most people. Relatedly, costs for healthcare were compounded by the need to travel outside of Lunga and many mentioned delaying healthcare treatment due to the costs.

For example, one person mentioned delaying seeking treatment because of prohibitive costs:

*“I didn’t go to facility earlier because I don’t have enough money for treatment.”*

Several participants mentioned bed nets being unaffordable:

*“Even if I want to buy, I can’t find it here. It’s being sold on Gambella around 400 to 500 [$7–8.8 USD]. Can you imagine going that far just to buy bed net?”.*

*“There is no one selling bed net, there is no supply even if there is a market, I can’t afford to buy [a net]”.*

*“No, we don't have [bed nets]. We can't neither afford nor find it easily. It is more than 300 Birr in Gambella.”*

#### Community support for those that are sick

Some people, particularly those involved in the gold mining industry, mentioned that there was a community support network to help sick people pay for and access treatment, but there were others that seemed unaware of this network.

*“Most mining workers suffer from malaria. So at that time we call the leader of the kebele and ask him to give us an ambulance then we collect some money and send him with one of his friends. If he still doesn’t feel better, he will be referred to Gambella for better treatment. We follow everything through phone and collect money from the community as needed. When he finally gets back here, we'll help him rest until he's healed.”*

## Discussion

The high prevalence rate among participants in this study shows that malaria is a significant public health problem for the community living at Lunga. Other studies within the Gambella Region have likewise indicated the general high burden of malaria in this setting, though published point prevalence surveys are scarce. One study found a *P. falciparum* seroprevalence of 65% (95% CI 58–71.4) [36]. Meanwhile other studies utilizing passive case detection have also found a high malaria burden for the woreda that Lunga is located in [31]. Malaria test positivity has also been high in other nearby areas within Gambella, such as Lare’s mean test positivity of 40.8% (2011–2021) [37]. Another study of asymptomatic pregnant women attending health clinics in Majang Zone in the Gambella Region found a *P. falciparum* prevalence of 15.3% (95% CI 12.1–18.9) [38]. Whereas a study, using multiple cross-sectional surveys, of asymptomatic people in different malaria transmission settings found an RDT/microscopy malaria prevalence of 23.7% (95% CI 16.9–32.3) and 34.2% (95% CI 26.1–43.4) by nPCR among people in Abobo (near where Lunga is located) [39].

As in other areas with high malaria burdens in this region, this study found that children under the age of five are at high risk for *P*. *falciparum* infections compared to adolescents and adults. Additionally, there is some geographic interplay with odds of malaria infection, as those originally from low-elevation areas of Ethiopia with high malaria test positivity, have indicated higher odds of malaria compared with those originally from higher elevation, low malaria test positivity, districts. Prior geographic location was also associated with bed net ownership, with those from higher elevation, low malaria test positivity areas being more likely to have and report using bed nets than those from lower elevation, high positivity areas. This study found household clustering of falciparum malaria and a logical association between not having a bed net to use and odds of testing positive for falciparum malaria. The qualitative interviews confirmed that many people in Lunga had moved to the location to work in gold mining. Conditions in the community are challenging. Participants mentioned a lack of affordable safe drinking water, sanitation systems, healthcare facilities, and schools.

Interestingly, despite the fact this study found that children under five were shown to be more at risk for malaria, few in Lunga knew that children under five could develop serious cases of malaria. This age group is at particular risk for severe disease and death from malaria infection [1, 40]. As such, this finding indicates a need to target households with young children in particular, for malaria education, prevention, and treatment interventions. In order to educate caregivers of young children about their risk for malaria and to provide these households with the means to protect against, and treat, this illness.

The results of this study have also highlighted a wider unmet need for malaria prevention and treatment, as well as general healthcare services, among the whole community living in Lunga. Most people surveyed knew bed nets could prevent malaria infections. Conversely, bed net use was very low (10%), likely because bed net ownership was low. For example, 51 out of 59 participants who reported having a bed net also reported using a bed net. This study also asked participants about bed net use in their households, and 95 out of 96 who reported no bed net use in their households reported that it was because they did not have access to bed nets. Low level bed net ownership among migrants have been found in other studies in Ethiopia too, 22.5% among migrant farm workers in one study in Amhara Region [10] and 11.9% in another study, also in Amhara Region [41]. This overall discordance between knowledge and net ownership is significant, given that this study’s findings also indicate that bed nets do have a strong protective effect against *P. falciparum* infections when they are utilized. Whilst bed net ownership does not always equate to utilization, among those in this study’s sample who had access to a bed net, utilization the night before was high (85%). As such, this indicates that increasing bed net ownership among this community has the potential to lower malaria transmission rates in Lunga.

Whilst some studies [42, 43] have found knowledge to be positively associated with bed nets in several settings, other studies [44–46] have found, akin to this study’s results, a discordance between knowledge and bed net ownership. A major cited reason for this discordance is lack of access, monetarily or in terms of physical access to bed nets [20, 44–47]. Similar findings were also found in a pilot study preceding this study at Lunga in 2020 [32] indicating little had changed within the two years between studies. Indeed, in this current study, participant interviews highlighted that low utilization was mainly due to the high cost of bed nets, which made them unaffordable, as well as lack of physical access to bed nets within the community. Community members would need to travel far to other towns to purchase them.

In terms of malaria treatment, similar dynamics also prevent many community members from receiving timely medical care. Visiting a doctor for any health need, including malaria symptoms, is often delayed due to lack of funds for treatment and requires long and costly travel to other towns.

Lack of material access to malaria prevention devices can, therefore, help to explain the low-level bed net utilization and delayed health-seeking behaviour at Lunga, despite knowledge about their benefits. Theories of health behaviour, including the social practice model of Shove et al*.* [48], which argues that material access is a key tenant of a person’s health ‘practices’, alongside competence (including knowledge) and meanings (socio-cultural expectations). Without one of the three (practices, competence, meanings), it is significantly harder for people to pursue positive health behaviours [48]. People living in Lunga are unable to actualize their knowledge of malaria prevention strategies due to limited material conditions. This indicates an unmet health need that should be addressed by public health interventions.

Other studies have focused on communities of migrants in Ethiopia that have found similar issues related to healthcare accessibility exacerbated by cost and distance [10, 41]. However, these communities often consist of short-term migrants working on large agricultural or other developmental projects, where there is an overall employer or organizer for the work migrants conduct. As such, frequently posed solutions to limited healthcare accessibility in these settings have been to involve the employing organization in the delivery of affordable and accessible health service. However, those living at Lunga do not have the option of accessing healthcare services organized or paid for by their employers, as most people moved independently to Lunga. Instead, other avenues for healthcare delivery will need to be considered in this setting, most likely involving private partnerships or an expansion of the local region’s primary healthcare network to cover this community.

Other research indicates that the legal precariousness of many gold mining communities in particular can exacerbate health and service accessibility even in the long term [20]. Such communities often operate outside of the traditional healthcare systems not just because of geographic isolation, but also due to community fear of ‘being found out’ preventing regular attendance at formal healthcare facilities [20, 45], or due to formalized structural barriers preventing community members from accessing health resources. As such, the findings of this research with regards to malaria risk and healthcare accessibility may persist over time in the absence of systematic interventions to target the unmet health needs of these communities.

This study has several limitations. One limitation is that the sampling strategy utilized an approach to estimate population prevalence of malaria but may not have been large enough to detect statistically significant differences in malaria prevalence between some demographic groups. For example, there were few people who were newly arrived at Lunga compared to those who had spent a longer period of time in the community. This could have impacted the analysis on potential associations between the duration of time spent living in Lunga and malaria infection.

This study detected malaria infections using RDTs and microscopy, which almost certainly missed some low parasite density infections. A more sensitive approach based on nucleci acids likely would have uncovered even more infections. This study’s prevalence estimates are, therefore, likely under-estimates. Furthermore, histidine-rich protein-2 (HRP2) and histidine-rich protein-3 (HRP3) gene deletions are known to be widespread in at least some parts of Ethiopia, potentially limiting the effectiveness of RDTs based on HRP2 [49]. As such, utilization of HRP2 RDTs likewise has the potential to underestimate prevalent malaria infections.

Additionally, as a cross-sectional design, this study was only able to capture the malaria disease burden and other individual, household or community attributes at the point of data collection. As an emerging community, there may be rapid changes occurring with regards to malaria risk and healthcare accessibility that were not captured in this research study. Having more detailed information about the exact living conditions, vector ecology and exposure, in places of origin would likewise have been valuable for the study. However, while the findings from this cross-sectional study may have limited generalizability, the observed patterns and dynamics between migration and health, including malaria, are likely to provide valuable insights for understanding similar issues in other newly settled communities. The specific context and characteristics of the population studied offer important lessons that could be applicable in comparable settings, even though temporal changes and differences in community conditions might affect the direct transferability of the results.

There are several strengths to this study as well. Foremost is the focus on a type of community that is frequently neglected in epidemiological research, for a variety of reasons. While such communities are frequently mentioned with regard to poor health outcomes, risk of spreading diseases across landscapes, and serving as a reservoir of disease, they often remain neglected in epidemiological research and in public health action. Finally, the study used a sampling strategy, structured questionnaire, and incorporated actual community-member voices in the analyses.

## Conclusions

Lunga is an example of an atypical community of settler migrants that is often acknowledged as neglected though important for infectious disease epidemiology. These types of settler populations living in emerging settlements, often based on extractive industries, may be growing, especially in Ethiopia. It is important to identify and address the health needs that these populations have. Conditions in these settlements may be challenging, exacerbating population health issues and hindering current accessibility to healthcare services. As such, interventions and plans for long-term service provision should be initiated to help resolve these communities’ unmet health needs.

Malaria was found to be a significant public health problem with a *P. falciparum* prevalence of 37.5% in the sample. Thus, considering this and the results of the analysis, public health interventions focusing on bed net distribution, perhaps especially among large households and those with children, are likely to be well-received and could have a major impact on the burden of malaria within the community. More generally, plans for getting sustained public health services in this, and similar, communities should be prioritized. Identifying these types of communities and their locations is a first step, followed by an establishment of their health needs and current accessibility to healthcare resources. Next, healthcare services should be provided to meet these needs. Ideally this would include establishing a health facility with affordable service provision within the community itself. All of these steps should include community participation to both identify and provide services that meet the needs of these communities.

## Supplementary Information

Supplementary Material 1.

## Data Availability

Data supporting the result are included within the article.
