# Supplementary material for: A cross-sectional study investigating malaria prevalence and associated predictors of infection among migrants to a newly established gold mining settlement in the Gambella Region of Ethiopia
Source: Malar J. 2024 Sep 30;23:292. doi: 10.1186/s12936-024-05117-4 (PMC11443954; doi:10.1186/s12936-024-05117-4)
Supplement: Supplementary file 1 — Supplementary Material 1. [file 12936_2024_5117_MOESM1_ESM.docx]

**Supplemental materials:**

**Supplemental Figure 1: Plot of malaria test positivity (number of positive tests divided by total number of tests) and mean elevation. Higher elevation woredas tended to have lower malaria test positivity.**

**
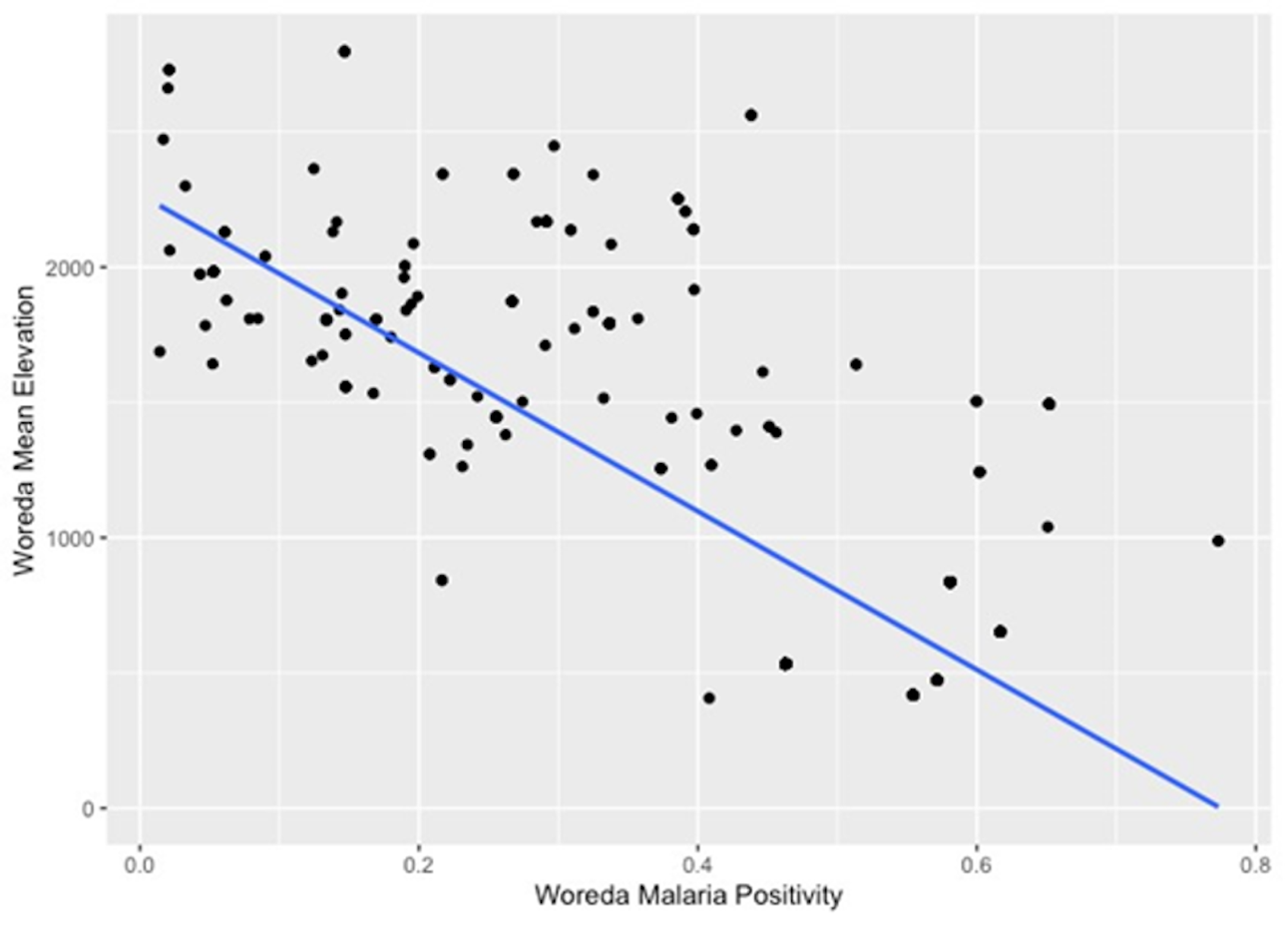
**

| **Variable 1** | **Variable 2** | **Spearman’s rho** | **p-value** |
| --- | --- | --- | --- |
| Mean Elevation | Malaria Positivity | -0.6034064 | <0.001 |

**Supplemental Figure 2: Knowledge Attitudes and Practices (KAP) Questionnaire**

| **Gambella 2022 Knowledge Attitudes and Practices Survey** | |
| --- | --- |
| Household Unique Identifier (HID) |  |
| Participant ID (PID) |  |
| GPS Longitude for House |  |
| GPS Latitude for House |  |
| **Participant Demographics** | |
| What is your age? |  |
| What is your gender? | Male  Female  Other |
| Are you currently pregnant? | Yes  No |
| What is your education level? | Cannot read or write  Can read or write but has no formal education  1 year of education  2 years of education  3 years of education  4 years of education  5 years of education  6 years of education  7 years of education  8 years of education  9 years of education  10 years of education  11 years of education  12 years of education  13 years of education  14 years of education  15 years of education or more  Other [specify] |
| Please specific “Other” |  |
| What is your religion? | Muslim  Orthodox  Protestant  Other [specify] |
| Please specific “Other” |  |
| What is your ethnicity? | Oromo  Ahmara  Agnuak  Yam  Dawuro  Other [specify] |
| Please specific “Other” |  |
| What is your main occupation/economic activity? | Farmer  Merchant  Government employment  Private Business  Other [specify] |
| Please specific “Other” |  |
| What is your marital status? | Unmarried  Married and living together Married and living separately Divorced  Widowed  Other [specify] |
| Please specific “Other” |  |
| How long [in months] have you lived in Lunga? |  |
| Where did you live in the year prior to moving to Lunga? [specify Region, Zone, Woreda, Kebele, Village] |  |
| How long did you live in this place before you moved to Lunga? |  |
| What are the two nearest neighboring villages to the location you lived in in the year prior to moving to Lunga? |  |
| Prior to moving to Lunga, where did you live for the longest amount of time? [specify Region, Zone, Woreda, Kebele, Village] |  |
| How long did you live in this place? |  |
| Where did you live for the longest amount of time when you were a child? [specify Region, Zone, Woreda, Kebele, Village] |  |
| How long did you live there when you were a child? |  |
| Why did you decide to move to Lunga? | Family ties  Economic opportunity in Lunga  No opportunities in previous location Other [specify] |
| Please specify "Other" |  |
| How did you find out about the opportunities at Lunga? |  |
| **Knowledge of Malaria** | |
| Have you ever heard of, or know of, an illness called malaria? | Yes  No |
| Can you tell me the main signs or symptoms of malaria? | Fever  Feeling cold  Headache  Nausea and Vomiting  Dizziness  Loss of appetite/refuse to eat or drink  Body ache or joint pain  Body weakness  Other [specify]  Don't Know  (Do NOT read options. Probe once. Can be multiple answers) |
| Please specify "Other" |  |
| In your opinion, what causes malaria? | Mosquito bites  House fly  Eating maize  Eating sugarcane  Hunger (empty stomach)  Eating/Drinking other dirty food/water Getting soaked with rain  Cold or changing weather Witchcraft  Other [specify]  Don't Know  (Do NOT read options. Probe once. Can be multiple answers) |
| Please specify "Other" |  |
| How can someone protect themselves against malaria? | Sleep under a mosquito net Mosquito repellent Applying skin lotion Smoking  Spraying house with insecticide  Keep house surroundings clean  Fill in puddles (stagnant water)  Burying or destroying cans and tins in pits Using window screens  Don't get soaked with ran Other [specify]  Don't Know  (Do NOT read options. Probe once. Can be multiple answers) |
| Please specify "Other" |  |
| In your opinion, who is most likely to get a serious case of malaria? [Choose one or two groups] | Adult men  Adult women Pregnant women  A six year old child  A three year old child  Don't Know  (Read options. ) |
| **Knowledge of Mosquito** | |
| Have you ever heard of an insect called the "mosquito"? | Yes  No |
| Where do you think mosquitoes come from? | Anywhere  Stagnant water  Vegetation/Grass  Swampy area  Other [specify]  Don’t know |
| Please specify "Other" |  |
| When do you think people get bite from mosquitos? | Daytime  Early evening  Late evening  Midnight  Early morning  Late morning  Other [specify]  Don't know |
| Please specify "Other" |  |
| Do you think people get mosquito bites outside? | Yes  No  Don’t Know |
| Do you think mosquito bites transmit malaria? | Yes  No  Don’t Know |
| Do you think outdoor mosquito bites can transmit malaria? | Yes  No  Don’t Know |
| **Practices of Malaria Prevention** | |
| What do you do to stop mosquito bites? | Bed nets  Mosquito repellent  Clothing  Opening of water drains  Coils  Inseciticides (sprays)  Burning organic materials  Screening windows and eaves  Other [specify]  None of these |
| Please specify "Other" |  |
| Do you have a mosquito net for personal use? | Yes  No |
| Did you use the mosquito net last night? | Yes  No |
| What were the reasons for not using the net last night? | Difficulty in hanging the net  Don't know how to use the net  Net isn't treated  It is too hot  Family member uses it instead  Other [specify] |
| Where is the easiest place you can get mosquito bed nets? | Local market  Friends  Health Post  Private Clinic  Pharmacy  Employer |
| **Healthcare Access** | |
| What mode of transport do you usually take to visit the nearest health facility? | Walk  Car  Motorcycle  Other [specify] |
| Please specify "Other" |  |
| When you walk to the nearest health facility, how long [in minutes] does it take you to get there? |  |
| On a scale of 1-5, where 1 is very easy and 5 is very difficult, how difficult is it to get medicine to treat your illness, when you, or a household member, is sick? | 1 [Very Easy]  2  3  4  5 [Very Difficult] |
| On a scale of 1-5, where 1 is very easy and 5 is very difficult, how difficult is it to go to a health facility when you, or a household member, is sick? | 1 [Very Easy]  2  3  4  5 [Very Difficult] |
| What type of place do you go to most often when you, or a household member, are sick? | Health Post  Health Center  Hospital  Pharmacy  Private Clinic  Other [specify] |
| Please specify "Other" |  |
| Is there anything that will stop you from easily getting to this place? | Yes  No |
| What will stop you from easily getting to the place  you go to when you, or a household member, are sick? | No money  No transportation  No time to go  No resources at the health facility Other [specify] |
| Please specify "Other" |  |
| **Care-seeking Behaviour** | |
| The last time you were sick with a fever, did you go to a health facility after your symptoms started? | Yes  No |
| How many days after your symptoms started was it before you went to a health facility? |  |
| The last time your child was sick with a fever, did you or someone else take them to a health facility after symptoms started? | Yes  No  [Do not fill in the question if you do not have a child] |
| How many days after symptoms started was it before you or someone else took your child to a health facility? |  |
| **Household Characteristics - Only the Head of Household to fill in this section. If participant is not the head of household then go to the next section (RDT).** | |
| How many people normally live in this household? | [usual household size, permanent members only] |
| How many children [under 18 years of age] live in this household? |  |
| How many children under five years of age live in this household? |  |
| How many pregnant people live in this household? |  |
| What type of wall was mainly used for the construction of this house? | Mud/wood  Mud/wood/stone  Wood  Stone  Block  Brick Cement/Plastered  Painted  Other [specify]  (Observation ) |
| Please specify "Other" |  |
| At any time in the past 12 months, has anyone sprayed the interior walls of your dwelling against  mosquitoes? | Yes  No  Don’t Know |
| How many months ago were the interior walls of your dwelling sprayed? |  |
| At any time in the past 12 months, have the walls in your dwelling been plastered or painted? | Yes  No  Don’t Know |
| How many months ago were the walls in your dwelling plastered or painted? |  |
| Does the house have eaves? | Yes  No |
| Are the eaves open? | Yes  No |
| What is the main source of water for domestic use for your household? | Piped into house  Piped outside the house  Buy from water vendors  Fetch from the river/stream  Borehole/Well  Other [specify] |
| Please specify "Other" |  |
| Does this house have any type of livestock? | Yes  No  [including hens] |
| Where are the animals kept at night? | Inside the house but separate from sleeping area for people  Inside the house in the same room as sleeping area for people  Outside the house/Open Space/Pens Other [specify] |
| Please specify "Other" |  |
| What animals are kept inside the house but separate from sleeping area for people? |  |
| What animals are kept inside the house in the same room as the sleeping area for people? |  |
| How many mosquito nets does your household have? |  |
| Were any mosquito nets used in your household last night? |  |
| How many mosquito nets were used in your household last night? |  |
| If your household has at least one bed net but the bed net was not used last night, what is the main reason for not using a mosquito net last night? | Difficulty in hanging the net  Don't know how to use the net  Net is not treated  It is too hot  Other [specify]  (Only answer if own at least one bed net) |
| Please specify "Other" |  |
| Was there any person who has been sick with fever in the household during the last 2 weeks? | Yes  No |
| **RDT Result Information** | |
| S.no |  |
| RDT Result |  |
| RDT species |  |
| **Microscopy Result Information** | |
| Microscopy Result |  |
| Microscopy species |  |
| Asexual counts [/200WBC] |  |
| Gametocyte counts [/200WBC] |  |

**Supplemental Figure 3: Qualitative Interview Guide**

Project: A mixed methods investigation into migration and malaria in an informal gold-mining settlement in Gambella Region of Ethiopia

Interviewer name:____________________

Interviewee ID:_______________________

Date (YYYY/MM/DD):__________________

1. What sources of information led you to know about Lunga?
2. How did you arrive in Lunga?
3. How do you make a living here in Lunga? (how do you obtain food and other necessities?)
4. If you have had malaria before, can you describe the experience? If you’ve had it multiple times, please describe the most recent episode.
   1. How long were you ill?
   2. How did you deal with the illness?
   3. Did anyone help you during your illness?
5. Please describe any efforts you take to prevent disease (from malaria or otherwise)
6. Please describe what you do if you become ill in Lunga

**Supplemental Figure 4(a)(b): Model 1A Spline Variable Plots.** The spline plot on the left indicates the interaction term between elevation of the place (woreda) of origin for an individual and the malaria test positivity (number of malaria positive tests divided by the number total tests) in that location (from 2021 – 2022). The interaction is depicted as a contour plot, with the values representing log-odds of falciparum infection, darker shades indicating a negative association and lighter shades indicating a positive association. Individuals from areas with higher test positivity and lower mean elevation were more likely to test positive for falciparum malaria (indicated by lighter colours and larger numbers in the contour plot) while those from low test positivity and high elevation settings were less likely to test positive (indicated by darker values and smaller numbers in the contour plot).

The spline function on the right is for the duration of time spent in Lunga (in months) and no detectable association was found (the line is flat on the zero point of the y-axis and the confidence intervals are wide).

**
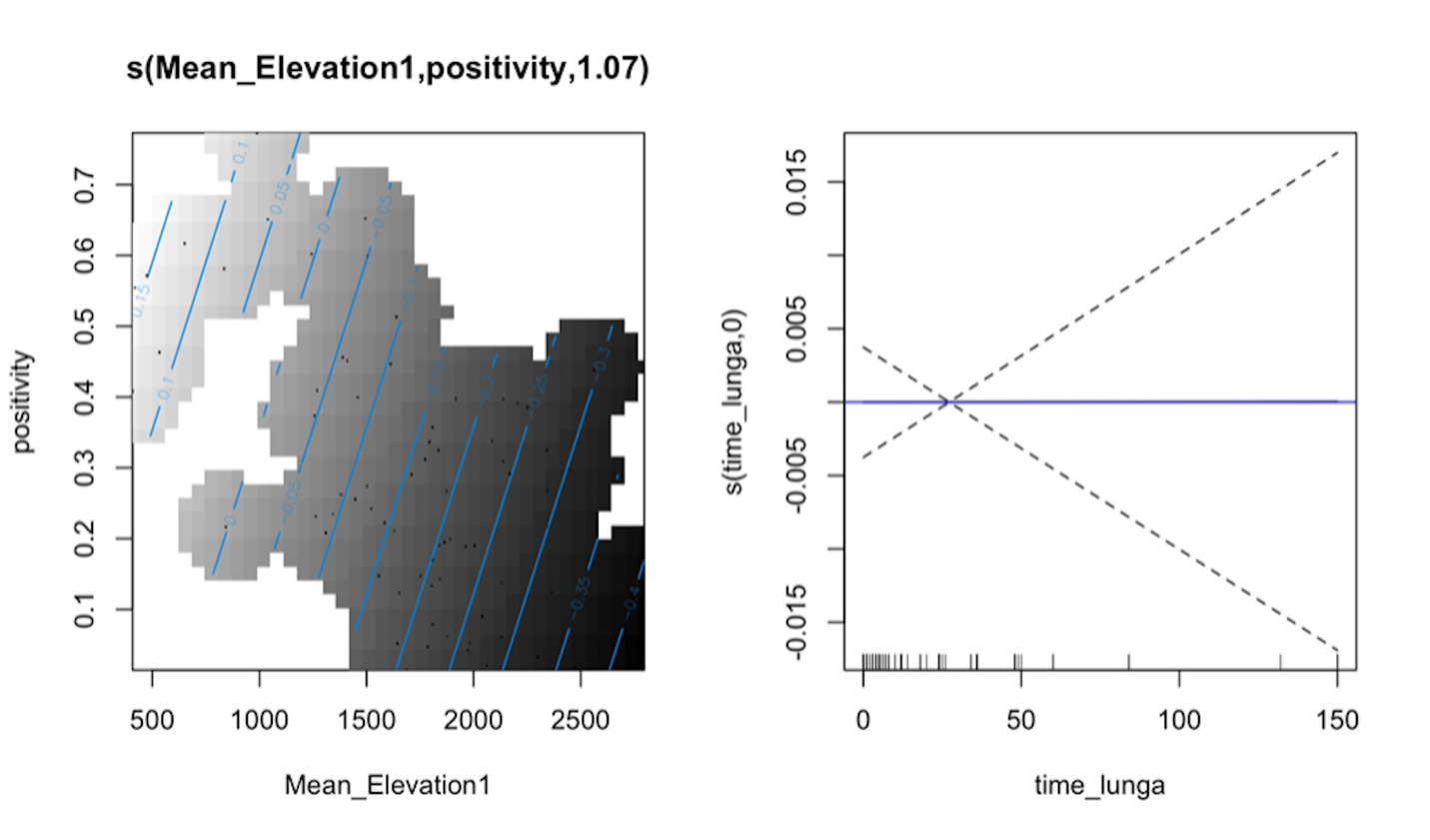
**

**Supplemental Figure 5(a)(b)(c): Model 1B Spline Variable Plots.** Three spline function plots are presented. The one on the far left indicates the interaction term between the number of individuals living in a house and the number of individuals in a house with a concurrent falciparum infection. Darker colours indicate negative associations while lighter colours indicate positive associations. Log-odds values are also presented in the contour lines. The highest association with odds of having a falciparum infection came from individuals who lived in houses with higher numbers of people approximately 15 (from the y-axis) and with large numbers of those individuals having concurrent falciparum infections (approximately 5 - 10 household members, from the x-axis).

The spline plot in the middle indicates the interaction term between elevation of the place (woreda) of origin for an individual and the malaria test positivity (number of malaria positive tests divided by the number total tests) in that location (from 2021 – 2022). As with the first model (Model 1A), individuals from areas with higher test positivity and lower mean elevation were more likely to test positive for falciparum malaria (indicated by lighter colours and larger numbers in the contour plot) while those from low test positivity and high elevation settings were less likely to test positive (indicated by darker values and smaller numbers in the contour plot).

The spline function on the right is for the duration of time spent in Lunga (in months) and no detectable association was found (the line is flat on the zero point of the y-axis and the confidence intervals are wide).

**
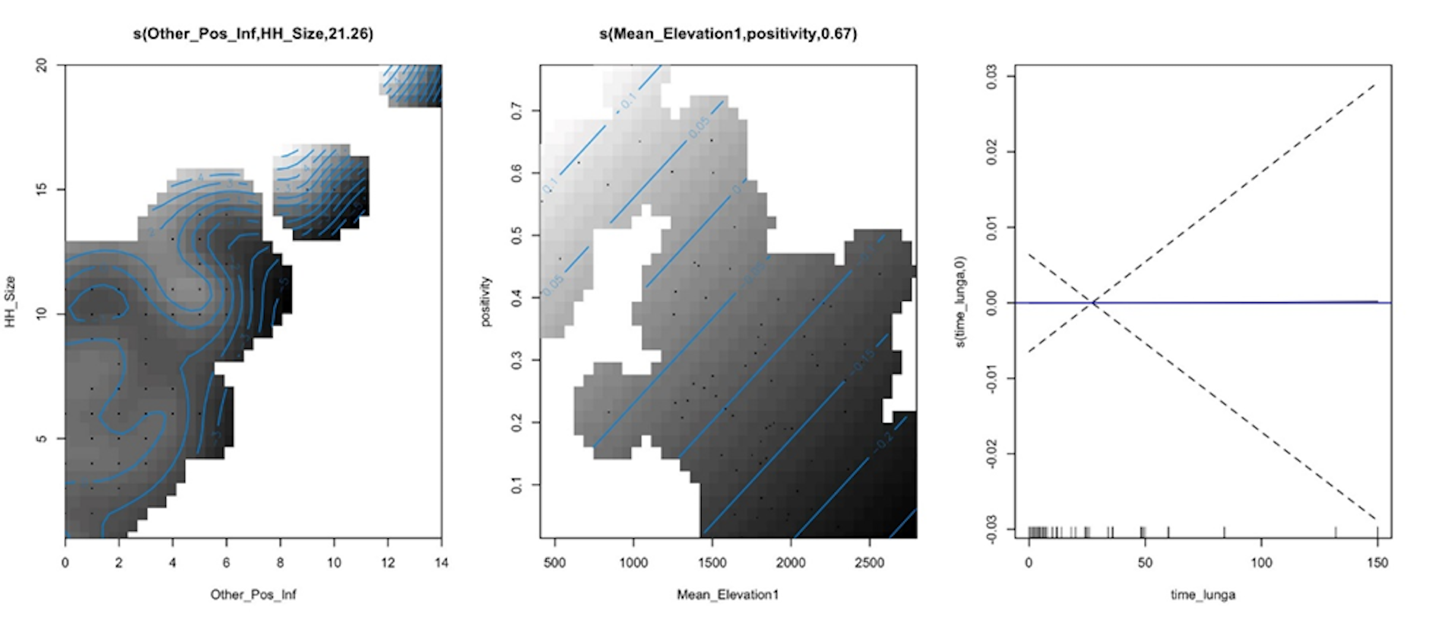
**

**Supplemental Figure 6(a)(b): Model 2A Spline Variable Plots.** The spline plot on the left indicates the interaction term between elevation of the place (woreda) of origin for an individual and the malaria test positivity (number of malaria positive tests divided by the number total tests) in that location (from 2021 – 2022). Individuals from areas with higher test positivity and lower mean elevation were less likely to own a bed net (indicated by darker colours and smaller numbers in the contour plot) while those from low test positivity and high elevation settings were more likely to own a bed net (indicated by lighter values and higher log-odds numbers in the contour plot).

The spline function on the right is for the duration of time spent in Lunga (in months) and no detectable association was found (the line is flat on the zero point of the y-axis and the confidence intervals are wide).

**
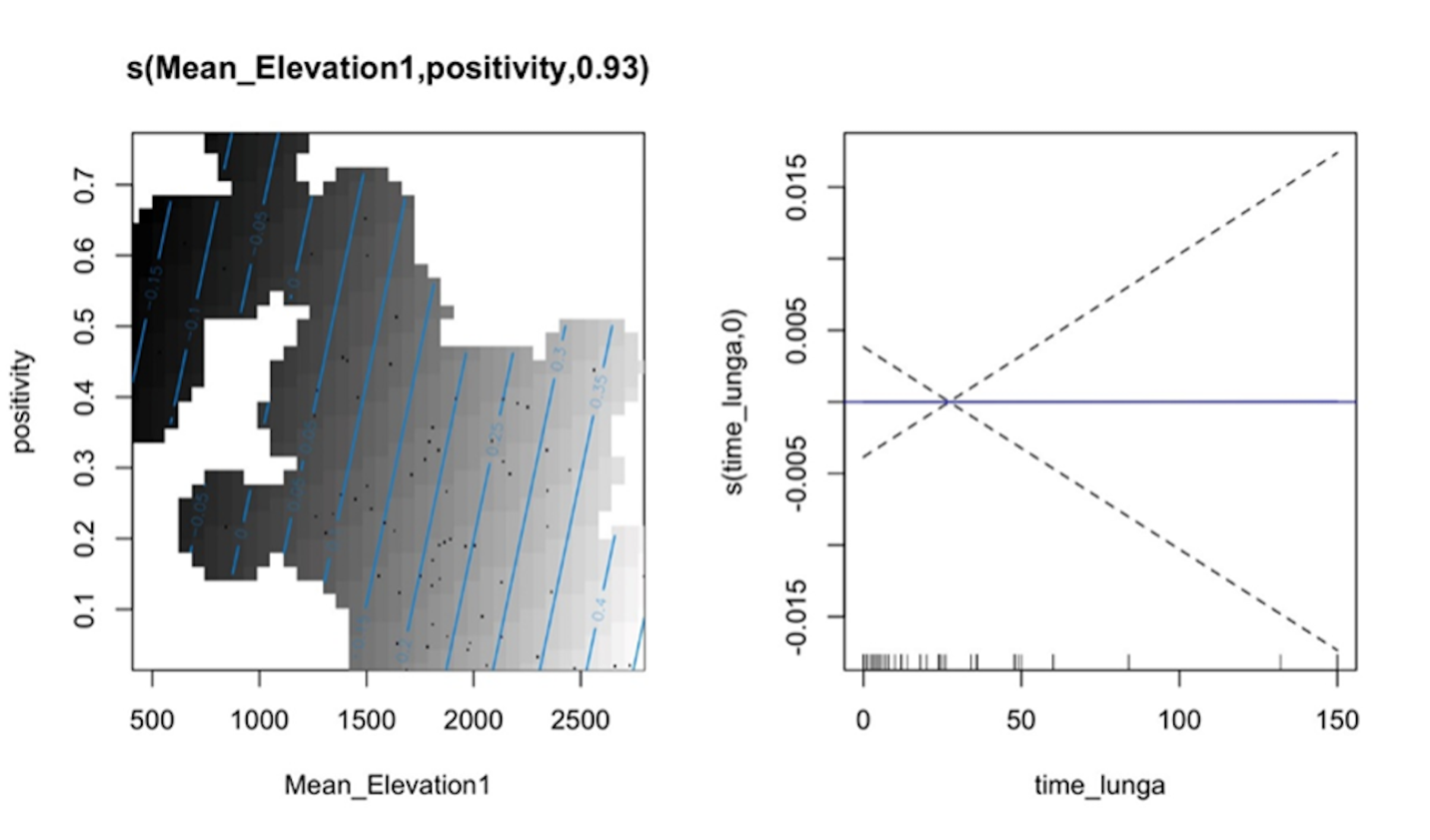
**

**Supplemental Figure 7(a)(b)(c): Model 2B Spline Variable Plots.** The spline plot on the left indicates the interaction term effect for the number of people in a household (y-axis) and the number of people in the household with a falciparum infection (x-axis) and the odds of an individual having a bed net. Individuals living in houses with large numbers of people (e.g. 15 people) and with large numbers of those household members being infected (e.g. 10 or more) were more likely to report not owning a bed net.

The spline plot in the middle indicates the association between interaction term between elevation of the place (woreda) of origin for an individual and the malaria test positivity (number of malaria positive tests divided by the number total tests) in that location (from 2021 – 2022) on the odds of having a bed net. Individuals from areas with higher test positivity and lower mean elevation were less likely to own a bed net (indicated by darker colours and smaller numbers in the contour plot) while those from low test positivity and high elevation settings were more likely to own a bed net (indicated by lighter values and higher log-odds numbers in the contour plot).

The spline function on the right is for the duration of time spent in Lunga (in months) and no detectable association was found (the line is flat on the zero point of the y-axis and the confidence intervals are wide).

**
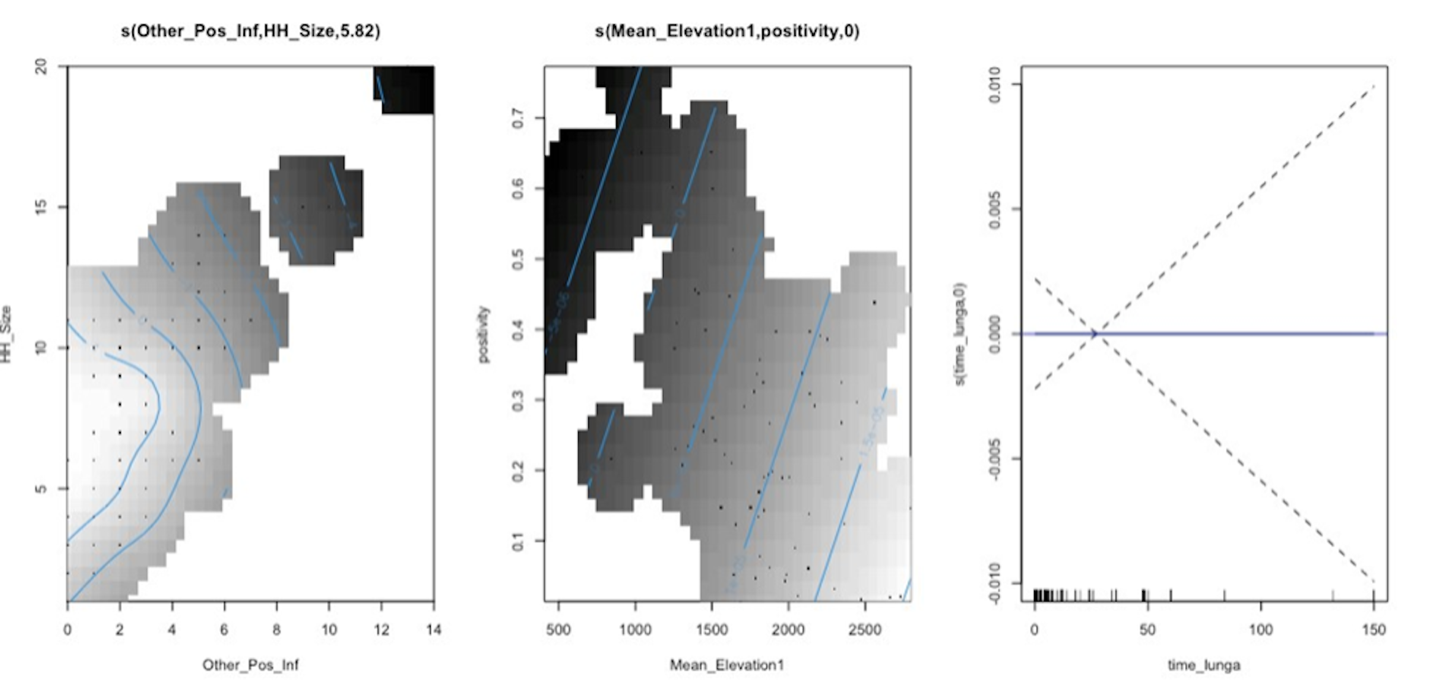
**

**Supplemental Table 1: Table of Variables**

| **Table of Variables** | | |
| --- | --- | --- |
| **Variable** | **Description** | **Variable Type in Model** |
| PF Status | The malaria *P. falciparum* status of an individual. An individual is positive following a positive test result on either an RDT or through microscopy testing.  Positive infection = 1  Negative infection = 0 | Binary variable/Outcome variable |
| Bed net | Whether a person has personal access to a bed net within their household  Yes = 1  No = 0 | Binary variable / Outcome variable |
| Age | The age of participants in four categories:  1-4 years of age  5-14 years of age  15-24 years of age  25+ years of age | Categorical variable |
| Gender | Reported gender of participants in two categories:  Male = 1  Female = 0 | Binary variable |
| Household Size | The number of people reported living in a household | Spline function |
| Other Household PF infections | The number of other positive cases of PF in a household excluding the PF status of the individual | Spline function |
| Time in Lunga | Time in months that a person has spent living at Lunga | Spline function |
| Mean woreda Elevation | The mean elevation of the woreda that a person moved to Lunga from | Spline function |
| Mean woreda positivity | The mean malaria test positivity for PF infections (2021 and 2022) of the woreda that a person moved to Lunga from | Spline function |
| Household ID | ID number assigned to each household in the sample | Spline function |

**Supplemental Table 2: Demographics of sample**

|  | **n** | **%** | **Mean** | **Range** |
| --- | --- | --- | --- | --- |
| **Age (years)** | - | - | 23 | 1-62 years |
| **Age Groups** |  |  |  |  |
| 1-4 | 48 | 8.15% | - | - |
| 5-14 | 43 | 7.30% | - |  |
| 15-24 | 248 | 42.11% | - | - |
| 25+ | 250 | 42,45% | - | - |
| **Gender** |  |  |  |  |
| Male | 335 | 54.92% | - | - |
| Female | 255 | 41.80% | - | - |
| **Pregnancy** |  |  |  |  |
| Yes | 10 | 2.46% | - | - |
| No | 396 | 97.54% | - | - |
| **Religion** |  |  |  |  |
| Catholic | 5 | 0.87% | - | - |
| Muslim | 53 | 9.19% | - | - |
| Orthodox | 179 | 31.02% | - | - |
| Protestant | 339 | 58.75% | - | - |
| Waqefeta | 1 | 0.17% |  | - |
| **Education (among adults 18 years+)** |  |  | - |  |
| None | 32 | 6.74% | - | - |
| Some primary education only | 239 | 50.32% | - | - |
| At least some secondary education | 204 | 42.95% | - | - |
| **Occupation** |  |  |  |  |
| Dependent | 69 | 11.90% | - | - |
| Farming | 14 | 2.14% | - | - |
| Gold mining | 270 | 46.55% | - | - |
| Hospitality | 26 | 4.48% | - | - |
| Housework | 22 | 3.79% | - | - |
| Merchant | 49 |  | - | - |
| Private Business | 83 | 8.45% | - |  |
| Student | 32 | 5.52% | - | - |
| Unemployed | 1 | 0.17% | - | - |
| Other | 14 | 2.14% | - | - |
| **Time in Lunga** |  |  |  |  |
| Months spent in Lunga | - | - | 27 months | 0-300 months |
| **Household size** |  |  |  |  |
| Number of people in a household | - | - | 6 people | 1-20 people |

**Supplemental Table 3:** Model output of spline functions from generalized additive logistic regressions for falciparum (PF) malaria infections and bed net access. Interpretation of coefficients is best by visualizing the plots of smoothed effects (Supplemental Figure 4(a)(b) and Figure 5(a)(b)(c)). Variable names correspond to Table 1. Model A only included individual level variables. Model B included those same variables as well as the household level interaction term for other house members having a falciparum infection and for the number of other occupants in the house.

|  | **Model 1A** | | | | **Model 1B** | | | |
| --- | --- | --- | --- | --- | --- | --- | --- | --- |
|  | **edf** | **Ref.df** | **Chi sq** | **p-value** | **edf** | **Ref.df** | **Chi sq** | **p-value** |
| **s(Other Household PF Infections, Household Size)** | - | - | - | - | 21.26 | 29 | 59.45 | <0.0001 |
| **s(Mean woreda Elevation, Mean woreda positivity)** | 1.07 | 29 | 3.40 | 0.0491 | 0.67 | 29 | 1.25 | 0.1520 |
| **s(Time in Lunga)** | 0.0002 | 9 | 0 | 0.7312 | 0.0005 | 9 | 0 | 0.6021 |

**Supplemental Table 4:** Model output of spline functions from generalized additive logistic regressions for falciparum malaria (PF) infections and bed net access. Interpretation of coefficients is best by visualizing the plots of smoothed effects (Supplemental Figure 6(a)(b) and Supplemental Figure 7(a)(b)(c)). Variable names correspond to Table 1. Model A only included individual level variables. Model B included those same variables as well as the household level interaction term for other house members having a falciparum infection and for the number of other occupants in the house.

|  | **Model 2A** | | | | **Model 2B** | | | |
| --- | --- | --- | --- | --- | --- | --- | --- | --- |
|  | **edf** | **Ref.df** | **Chi sq** | **p-value** | **edf** | **Ref.df** | **Chi sq** | **p-value** |
| **s(Other Household PF Infections, Household Size)** | - | - | - | - | 5.82 | 29 | 21.51 | <0.0001 |
| **s(Mean woreda Elevation, Mean woreda positivity)** | 0.93 | 29 | 2.34 | 0.0869 | 0.0001 | 29 | 0 | 0.6122 |
| **s(Time in Lunga)** | 0.0001 | 9 | 0 | 0.7497 | 0.00001 | 9 | 0 | 0.9429 |
|  |  |  |  |  |  |  |  |  |

**Supplemental Table 5: KAP Model Results I**

|  | **Knowledge of mosquito bites causing malaria** | **Knowledge of sleeping under a bed net** | **Knowledge of mosquito repellent** | **Knowledge of household insecticide spraying** | **Pregnant people can get serious case of malaria** | **Children under 5 can get serious case of malaria** |
| --- | --- | --- | --- | --- | --- | --- |
| **Age** | 0.95  (0.91-0.99) | 0.96  (0.92-1.00) | 0.97  (0.90-1.06) | 0.97  (0.93-1.02) | 0.99  (0.96-1.02) | 1.04  (1.00-1.07) |
| **Gender** |  |  |  |  |  |  |
| Female (reference) | - | - | - | - | - | - |
| Male | 3.11  (1.23-7.86) | 1.70  (0.78-3.73) | 0.26  (0.07-0.98) | 1.83  (0.74-4.54) | 0.90  (0.50-1.64) | 0.73  (0.41-1.30) |
| **Occupation** |  |  |  |  |  |  |
| No Gold mining | - | - | - |  | - | - |
| Yes Gold mining | 0.32  (0.12-0.85) | 0.60  (0.27-1.33) | 1.27  (0.39-4.09) | 0.92  (0.36-2.38) | 0.74  (0.41-1.31) | 1.11  (0.63-1.94) |
| **Education** |  |  |  |  |  |  |
| None (reference) | - | - | - | - | - | - |
| Primary | 1.14  (0.31-4.15) | 1.98  (0.70-5.60) | 0.42  (0.09-2.02) | 1.62  (0.36-7.39) | 0.84  (0.28-2.50) | 1.51  (0.57-4.03) |
| Secondary | 1.60  (0.41-6.27) | 2.44  (0.82-7.23) | 0.91  (0.20-4.17) | 1.73  (0.37-8.20) | 1.05  (0.35-3.18) | 1.45  (0.54-3.90) |
|  |  |  |  |  |  |  |
| **AIC** | 234.5062 | 310.9768 | 155.824 | 236.9733 | 441.4158 | 482.5724 |
| **n** | 421 | 421 | 421 | 412 | 421 | 421 |

**Supplemental Table 6: KAP Model Results II**

|  | **Fever** | **Feeling cold** | **Headache** | **Nausea and vomiting** | **Dizziness** | **Loss of appetite** | **Body ache/joint pain** | **Body weakness** |
| --- | --- | --- | --- | --- | --- | --- | --- | --- |
| **Age** | 0.99 (0.95-1.03) | 0.96 (0.93-0.99) | 0.99 (0.96-1.03) | 1.02  (0.99-1.05) | 1.02  (0.98-1.05) | 1.02  (0.98-1.07) | 0.99  (0.94-1.04) | 0.98  (0.94-1.02) |
| **Gender** |  |  |  |  |  |  |  |  |
| Female (reference) | - | - | - | - | - | - | - | - |
| Male | 1.37 (0.65-2.87) | 1.18 (0.66-2.14) | 1.37 (0.76-2.47) | 0.59  (0.33-1.03) | 0.39  (0.21-0.74) | 0.35  (0.16-0.77) | 0.98 (0.47-2.05) | 1.20 (0.62-2.33) |
| **Occupation** |  |  |  |  |  |  |  |  |
| No Gold mining | - | - | - | - | - | - | - | - |
| Yes Gold mining | 0.66 (0.32-1.34) | 0.75 (0.41-1.35) | 1.37 (0.75-2.31) | 2.00  (1.15-3.51) | 2.64  (1.38-5.08) | 2.74  (1.25-6.03) | 1.00  (0.49-2.07) | 0.74 (0.39-1.39) |
| **Education** |  |  |  |  |  |  |  |  |
| None (reference) | - | - | - | - | - | - | - | - |
| Primary | 0.85 (0.26-2.81) | 1.51 (0.59-3.88) | 1.43 (0.61-3.33) | 2.06  (0.71-5.98) | 2.03  (0.53-7.76) | 2.71  (0.33-22.11) | 0.62 (0.18-2.13) | 1.24 (0.38-4.04) |
| Secondary | 1.26 (0.37-4.30) | 1.30 (0.49-4.45) | 2.40 (0.99-5.85) | 2.70  (0.93-7.88) | 4.16  (1.09-15.85) | 3.09  (0.38-25.22) | 0.68  (0.20-2.34) | 0.97 (0.29-3.23) |
|  |  |  |  |  |  |  |  |  |
| **AIC** | 325.3193 | 483.3765 | 474.1287 | 500.9739 | 422.5835 | 277.3923 | 306.1645 | 372.2643 |
| **n** | 421 | 421 | 421 | 421 | 421 | 421 | 421 | 421 |
